# Limitations of Artificial Intelligence in Plastic Surgery

**DOI:** 10.1093/asj/sjad357

**Published:** 2023-11-28

**Authors:** Ravi Dhawan, Kendall Douglas Brooks

We read with great interest the recent informative article by Palacios and Bastidas on the applications of artificial intelligence (AI) in the field of plastic surgery, with possibilities such as copywriting, chatbots, and research. However, we believe there are essential facets of the topic that merit further consideration.^1^

While AI's role in assisting tasks like copywriting and research is undeniable, its potential to directly influence patient outcomes and clinical outcomes is vast. Plastic surgery, like many other medical fields, relies heavily on visual outcomes. The potential for AI to interpret medical images may be transformative. Of particular interest to plastic surgery is the ability of AI to analyze human photographs. With advanced machine learning algorithms, AI can evaluate and quantify results of cosmetic and reconstructive surgeries, offering objective assessments that can complement a surgeon's subjective evaluation. This could provide significant value to preoperative counseling, operative planning, and following postoperative outcomes.^2^ Even ChatGPT's (OpenAI, San Francisco, CA) non-HIPAA compliant recent GPT-4 update includes image analysis capabilities.^3^

Research could include deeper exploration of AI's capabilities for clinical decision-making, preoperative planning, and postoperative care in the context of aesthetic plastic surgery.^2,4,5^ AI may become an invaluable asset in predicting surgical outcomes, determining the most efficient procedures, and even monitoring postoperative recovery.^4^ However, it is important that the plastic surgery profession carefully consider the ethical concerns surrounding AI and its integration into practice. Given that the field often grapples with societal standards of beauty, biased AI could potentially influence evaluations or recommendations. Addressing these biases is paramount to ensuring equitable and unbiased patient care.

If we are to integrate AI beyond natural language process chatbots into plastic surgery practices, ethical considerations are paramount. As Palacios and Bastida suggested, biases in AI training data must not be overlooked. Moreover, with the proliferation of publicly available AI engines, HIPPA compliance is a particular concern. This brings into question how we may best help current and future surgeons adapt. Curricula would benefit from the inclusion of AI ethics, AI-augmented clinical techniques, and hands-on training with AI tools. Last, at the cornerstone of any medical intervention lies the trust between a patient and their healthcare provider, built upon empathy, transparency, and competence of the practitioner. Surgeons and researchers must consider the influence AI may have on patient trust and satisfaction, especially because applications of AI in plastic surgery are in the nascent stages, and there is limited understanding of AI's accuracy, reliability, and confidentiality.

The onus is on medical professionals to adapt alongside emerging technologies, ethically and safely integrating them into their practices in a manner that retains or improves patient experience and care. Over history, technological developments have reshaped healthcare and plastic surgery in ways once thought impossible. With the ongoing enhancements in technology, we may be standing on the brink of a pivotal shift in how care is planned, delivered, and monitored.
